# Progesterone stimulates histone citrullination to increase IGFBP1 expression in uterine cells

**DOI:** 10.1530/REP-21-0132

**Published:** 2021-05-25

**Authors:** Coleman H Young, Bryce Snow, Stanley B DeVore, Adithya Mohandass, Venkatesh V Nemmara, Paul R Thompson, Baskaran Thyagarajan, Amy M Navratil, Brian D Cherrington

**Affiliations:** 1 Department of Zoology and Physiology, University of Wyoming, Laramie, Wyoming, USA; 2 Department of Pediatrics, University of Cincinnati College of Medicine, Cincinnati, Ohio, USA; 3 University of Wyoming School of Pharmacy, Laramie, Wyoming, USA; 4 Department of Chemistry and Biochemistry, Rowan University, Glassboro, New Jersey, USA; 5 Department of Biochemistry and Molecular Pharmacology, University of Massachusetts Medical School, Worcester, Massachusetts, USA

## Abstract

Peptidylarginine deiminases (PAD) enzymes were initially characterized *in uteri*, but since then little research has examined their function in this tissue. PADs post-translationally convert arginine residues in target proteins to citrulline and are highly expressed in ovine caruncle epithelia and ovine uterine luminal epithelial (OLE)-derived cell line. Progesterone (P4) not only maintains the uterine epithelia but also regulates the expression of endometrial genes that code for proteins that comprise the histotroph and are critical during early pregnancy. Given this, we tested whether P4 stimulates PAD-catalyzed histone citrullination to epigenetically regulate expression of the histotroph gene insulin-like growth factor binding protein 1 (*IGFBP1*) in OLE cells. 100 nM P4 significantly increases IGFBP1 mRNA expression; however, this increase is attenuated by pre-treating OLE cells with 100 nM progesterone receptor antagonist RU486 or 2 µM of a pan-PAD inhibitor. P4 treatment of OLE cells also stimulates citrullination of histone H3 arginine residues 2, 8, and 17 leading to enrichment of the ovine *IGFBP1* gene promoter. Since PAD2 nuclear translocation and catalytic activity require calcium, we next investigated whether P4 triggers calcium influx in OLE cells. OLE cells were pre-treated with 10 nM nicardipine, an L-type calcium channel blocker, followed by stimulation with P4. Using fura2-AM imaging, we found that P4 initiates a rapid calcium influx through L-type calcium channels in OLE cells. Furthermore, this influx is necessary for PAD2 nuclear translocation and resulting citrullination of histone H3 arginine residues 2, 8, and 17. Our work suggests that P4 stimulates rapid calcium influx through L-type calcium channels initiating PAD-catalyzed histone citrullination and an increase in *IGFBP1* expression.

## Introduction

Different species share similar mechanisms for the establishment of pregnancy. Many species’ uterine luminal epithelial cells undergo dramatic reorganization to form glandular epithelia over the course of a female reproductive cycle (Ramathal *et al.* 2010, Spencer 2014). In sheep, knockout of uterine glands results in pregnancy loss and infertility (Gray *et al.* 2001, Spencer & Gray 2006, Filant & Spencer 2013). Uterine glandular epithelia secret numerous molecules, collectively known as the histotroph that are critical for the development and survival of the conceptus (Bazer 1975, Kane *et al.* 1997, Spencer 2014). One such histotroph molecule secreted by uterine epithelia is insulin-like growth factor-binding protein-1 (IGFBP1). During the secretory phase of the estrous cycle, elevated progesterone (P4) stimulates *IGFBP1* gene expression. In several species, including humans and sheep, IGFBP1 is critical for conceptus migration and placentation (Fowler *et al.* 2000, Simmons *et al.* 2009).

Some of the original studies investigating the peptidylarginine deiminase (PAD or PADI) enzyme family were conducted using rodent uteri (Takahara *et al.* 1989, 1992). More recent genome sequencing analysis confirms that PAD expression is higher in uteri than compared to the 50 other tissues examined (Barrett *et al.* 2009). In the presence of calcium, PADs convert positively charged arginine amino acids in target proteins into neutral citrulline residues, a reaction termed as 'citrullination' or 'deimination'. Early studies discovered that PAD expression in uteri is estrogen-dependent and that levels fluctuate across the estrous cycle (Takahara *et al.* 1992). In fact, one of the first cDNAs of a PAD enzyme was cloned from rodent uteri; however, since these groundbreaking studies, little work has investigated the functional role of PADs in this tissue (Takahara *et al.* 1989, 1992, Terakawa *et al.* 1991). Similar to rodent uteri, PADs are highly expressed in luminal and glandular epithelial cells in caruncle tissue from sheep during early pregnancy. We also found that PADs citrullinate arginine residues 2, 8, and 17 on the histone H3 tail to regulate basal expression of *IGFBP1* in an ovine luminal epithelial (OLE) cell line (Young *et al.* 2017). Given that *IGFBP1* expression is critical to maintain early pregnancy and is stimulated by P4, we investigated whether P4 induces histone citrullination to regulate *IGFBP1* expression.

Calcium influx and its effect on intracellular signaling are critical for adhesion, decidualization, and placentation (Lee *et al.* 2009, Kumar *et al.* 2015, De Clercq & Vriens 2018). It is also established that PAD enzymatic activity and nuclear translocation are calcium-dependent (Arita *et al.* 2004, Zheng *et al.* 2019); however, the mechanisms underlying P4-induced calcium influx and the link to PAD function are not clear. With classical steroid receptor signaling, P4 diffuses through the plasma membrane and binds to the nuclear progesterone receptor (nPR). The complex then translocates to the nucleus and binds progesterone response elements (PREs) in promoters to activate gene expression. Currently, there is limited evidence in the literature to suggest that the nPR is directly involved in mediating a rapid calcium influx into cells. Membrane progesterone receptors (mPRs) further complicate our understanding of a P4-induced calcium influx mechanism in uterine epithelial cells. Ovine mPRs bind P4 with high affinity and can modulate intracellular calcium levels (Ashley *et al.* 2006, 2009, Viero *et al.* 2006, Dressing *et al.* 2011). Additionally, unique P4-mediated mechanisms, independent of nPRs and mPRs, have also been discovered that trigger rapid calcium influx such as in human sperm cells and in *Xenopus laevis* oocytes (Wasserman *et al.* 1980, Thomas & Meizel 1989). Currently, how P4 induces calcium influx in uterine luminal epithelial cells is unclear.

Herein, our studies show that P4 stimulates citrullination of histone H3 arginine residues 2, 8, and 17 and results in a significant increase in IGFBP1 mRNA in OLE cells. The increase in IGFBP1 mRNA is inhibited by pretreating OLE cells with P4 antagonist RU486 and the pan-PAD inhibitor, BB-Cl-amidine (BB-ClA). We also demonstrate that citrullinated histones are enriched on the ovine *IGFBP1* gene promoter following P4 treatment compared to vehicle-treated controls. Interestingly, our results show that P4 initiates a rapid calcium influx through an L-type calcium channel, which is important for PAD2 nuclear translocation and ultimately histone citrullination in OLE cells. Collectively, our work suggests that P4 stimulates rapid calcium influx through L-type calcium channels initiating PAD-catalyzed histone citrullination and increased *IGFBP1* expression.

## Materials and methods

### Cell culture and materials

OLE cells, a generous gift from Dr Greg Johnson, were maintained in high glucose DMEM containing 2 mM glutamine, 100 U penicillin/mL, 100 μg streptomycin/mL and 10% fetal bovine serum (FBS) (HyClone, Logan, UT, USA) as previously described (Young *et al.* 2017). For all P4 (MiliporeSigma) treatments, OLE cells were incubated overnight in phenol red-free DMEM (Hyclone) with 2.5% FBS (Corning). All cells were grown in 5% CO_2_ at 37°C in a humidified environment. OLE cells were treated with PAD inhibitor, biphenyl-benzimidazole-Cl-amidine (BB-ClA) which was synthesized and generously provided by Dr Paul R Thompson as previously described (Knight *et al.* 2015).

### Ewe uterine epithelial cells

Primary ewe uterine epithelial cells were collected from dissected uterine horns as previously described (Johnson *et al.* 1999). Briefly, each uterine horn was washed with PBS and filled with 20 mL of Hanks’ Balanced Salt Solution (HBSS) with 2.5 mg/mL pancreatin and 4.8 mg/mL of dispase II (MiliporeSigma). Uterine horns were incubated at 37°C for 1 h and massaged to remove epithelial cell sheets. Cells were then dispersed, plated, and maintained in high glucose DMEM containing 2 mM glutamine, 100 U penicillin/mL, 100 μg streptomycin/mL and 10% FBS (HyClone). All cells were maintained in 5% CO_2_ at 37°C in a humidified environment. Rambouillet ewes (*Ovis aries*) were maintained with access to food and water *ad libitum*. Euthanasia and tissue harvest were performed in accordance with the guidelines outlined in the Report of the AVMA on Euthanasia. The study was approved by the University of Wyoming Institutional Animal Care and Use Committee (protocol #20190618BC00377-01).

### Immunocytochemistry (ICC)

Primary ewe uterine epithelial and OLE cells were plated in MatTek Life Sciences 35 mm glass-bottom dishes (Ashland, MA, USA). After being fixed and permeabilized, cells were incubated with primary antibodies overnight at 4°C (anti-H3Cit 2,8,17 1:150, Abcam ab5103), (anti-PAD2 1:150, Proteintech 12110-1-AP, Rosemont, IL, USA), (anti-PAD4 1:100, MiliporeSigma, P4749). Duplicate dishes were incubated with an equal mass of non-specific rabbit IgG as a negative control. The following morning dishes were washed 3× in PBS before incubation with a fluorophor-conjugated secondary antibody. Cells were imaged using a Zeiss 710 or 980 LSM confocal microscope under a 40× or 63× objective, respectively.

### Western blotting

OLE cells were treated with vehicle or 100 nM P4 for 30 or 60 min. For nicardipine studies, OLE cells were pre-treated with 10 nM nicardipine (MiliporeSigma) for 30 min followed by addition of vehicle or 100 nM P4 for an additional 30 (PAD2) or 60 min (H3Cit 2,8,17). OLE nuclear fractionation and histone purification were performed as previously described (Bezy *et al.* 2007, Shechter *et al.* 2007, Cherrington *et al.* 2012, Young *et al.* 2017). Protein concentration of OLE lysates and purified histones was measured by Pierce 660 nm protein assay (Thermo Fisher Scientific Inc.) prior to gel loading to ensure equal protein loading. Sample buffer consisting of 0.5 M Tris-HCl (pH 6.8), 60% glycerol, 30 mM DTT, 6% SDS was added to samples to yield a final concentration of 1× and then boiled at 95°C for 5 min. The samples were subjected to SDS-PAGE using 12 or 15% gels (acrylamide:bis-acrylamide ratio of 29:1) and subsequently transferred to Immobilon PVDF membranes (MilliporeSigma). Membranes were blocked in 1× casein (Vector Labs, Burlingame, CA, USA) diluted in TBS containing 0.1% Tween-20 (TBS-T) overnight at 4°C. Primary antibodies were incubated 1:1000 overnight at 4°C: H3Cit 2,8,17, PAD2, and total histone H3 (Abcam, ab1791). The following morning, membranes were washed in TBS-T, followed by a 2-h incubation at room temperature with 1:10,000 goat anti-rabbit HRP (Jackson ImmunoResearch Labs) secondary antibody. All blots were washed for 50 min (5 × 10 min) with TBS-T after secondary antibody incubation and then visualized using SuperSignal West Pico and Femto chemiluminescence substrate (Thermo Fisher Scientific Inc.). Quantitative densitometry analysis was conducted with Bio-Rad Image Lab software (Hercules, CA, USA). The experiments were repeated at least three independent times and values are expressed as the mean ± s.e.m. Means were separated by one-way ANOVA using SNK and * indicates significantly different means *P* < 0.05.

### qPCR

Total RNA was purified from OLE cells following 2 h treatment with vehicle or 1, 10, and 100 nM P4. For PAD inhibitor studies, OLE cells were pre-treated with 100 nM RU486 (MilliporeSigma) for 1 h or 2 µM BB-ClA for 3 h then stimulated with 100 nM P4 for an additional 2 h. Primary ewe uterine epithelial cells were also treated with 100 nM P4 for 2 h. RNA was purified according to the Omega Bio-Tek Total RNA Kit protocol (Omega Bio-Tek, Inc., Norcross, GA, USA). One microgram of resulting RNA was reverse transcribed using iScript RT Supermix for RT-qPCR (Bio-Rad). cDNA was subjected to real-time PCR analysis with SYBR Green (Bio-Rad) using intron spanning primers: IGFBP1 FWD 5′-CAGCAAACAGTGTGAGACTTCG-3′, REV 5′-TCCCACTCCAAGGGTAGACA-3′; GAPDH FWD 5′-CGTTCTCTGCCTTGACTGTG-3′, REV 5′-TGACCCCTTCATTGACCTTC-3′. Data were analyzed using the ΔΔct method in which ct values of target genes were adjusted to the corresponding ct value of the reference gene (GAPDH). The experiment was repeated at least three independent times and values are expressed as the mean ± s.e.m. Means were separated using SNK or a one-tailed paired Student’s *t*-test and * indicates significantly different means (*P < 0.05).

### Chromatin immunoprecipitation

Following overnight serum starvation, OLE cells were treated with either vehicle or 100 nM P4 for 1 h then cross-linked at room temperature (RT) in 1% formaldehyde for 10 min. ChIP was performed with a SimpleChip® Plus Enzymatic Chromatin IP Kit following the manufacturer’s protocol (Cell Signaling Technologies) and optimized for OLE cells. The chromatin was immunoprecipitated using anti-H3Cit 2, 8, 17 (Abcam), while non-specific IgG and total histone H3 are controls. Enriched DNA was then subjected to qPCR using primers that scan approximately 1000 bps upstream of the transcriptional start site of the ovine *IGFBP1* gene: 1 (-984/-717 bp) FWD 5′-GAGGCTGAAAGACAGAGGAAAC-3′, REV 5′-CCCAGTTAACAGAGCTTCCA-3′; 2 (−716/−509 bp) FWD 5′-CTTTGGGGGCTATGGTGAGAC-3′, REV 5′-AAGAGGAAGGAGCGCTTTGAA-3′; 3 (−511/−310 bp) FWD 5′-CTTCCCGGGCCTTGATTTC-3′, REV 5′-GTTCAGACCTGGAGCCAAAGT-3′; 4 (−300/−100 bp) FWD 5′-AGGACAAACACAGTCTGAAACG-3′, REV 5′-TGGCCGATGCTCGCTGA-3′. Levels of citrullinated histones associated with the ovine *IGFBP1* gene promoter were normalized to IgG using the fold enrichment method and are shown as vehicle compared to P4-treated cells for each of the four primer sets. The ChIP experiments were performed si independent times. Samples were analyzed using a one-tailed paired Student’s *t*-test in which vehicle was compared to P4-treated cells and * indicates significantly different means (*P < 0.05).

### Intracellular Ca^2+^ imaging

OLE cells were grown on 25 mm circular coverslips and incubated with the fluorescent Ca^2+^ indicator Fura-2AM (2 μM) for 1 h at RT in normal extracellular solution (NES) (137 NaCl mM, 5 mM KCl, 1 mM MgCl_2_, 2 mM CaCl_2_, and 10 mM HEPES and pH adjusted to 7.4 by NaOH). Coverslips were washed and incubated with NES for 30 min at RT in the dark. The coverslips were placed in a stainless-steel holder (bath volume ∼0.8 mL) and imaged using a Leica DMI300 B inverted microscope coupled to a TILL Polychrome V digital imaging system (Toptica Photonics, Farmington, NY, USA). Cells were treated with flow-through of 1 µM P4 for 100–400 seconds, 10 nM for 500–700 s, and 80 mM KCl at 800 s. In parallel, a second coverslip of cells was pretreated with 10 nM nicardipine for 30 min followed by the same treatment paradigm described previously. Results present the ratio (R/R_0_) of fluorescence intensities at the excitation wavelengths of 340 and 380 nm. Ca^2+^ imaging data were analyzed using Origin 2020 Software (Origin Lab, Northampton, MA, USA), and data for all figures are expressed as mean ± s.e.m. Statistical significance was calculated using one-way ANOVA followed by Student’s *t-*test and ** represents statistical significance (**P < 0.01).

### Statistical analysis

All experiments were repeated independently at least three times and resulting values are expressed as the mean ± s.e.m. Statistical analysis was determined using GraphPad Prism 6.0 (GraphPad Software). Means were separated using SNK or Student’s *t*-test and * indicate significantly different means (*P < 0.05 and **P < 0.01).

## Results

### Progesterone stimulates IGFBP1 mRNA expression in OLE and ovine primary uterine epithelial cells

**Figure 1:**
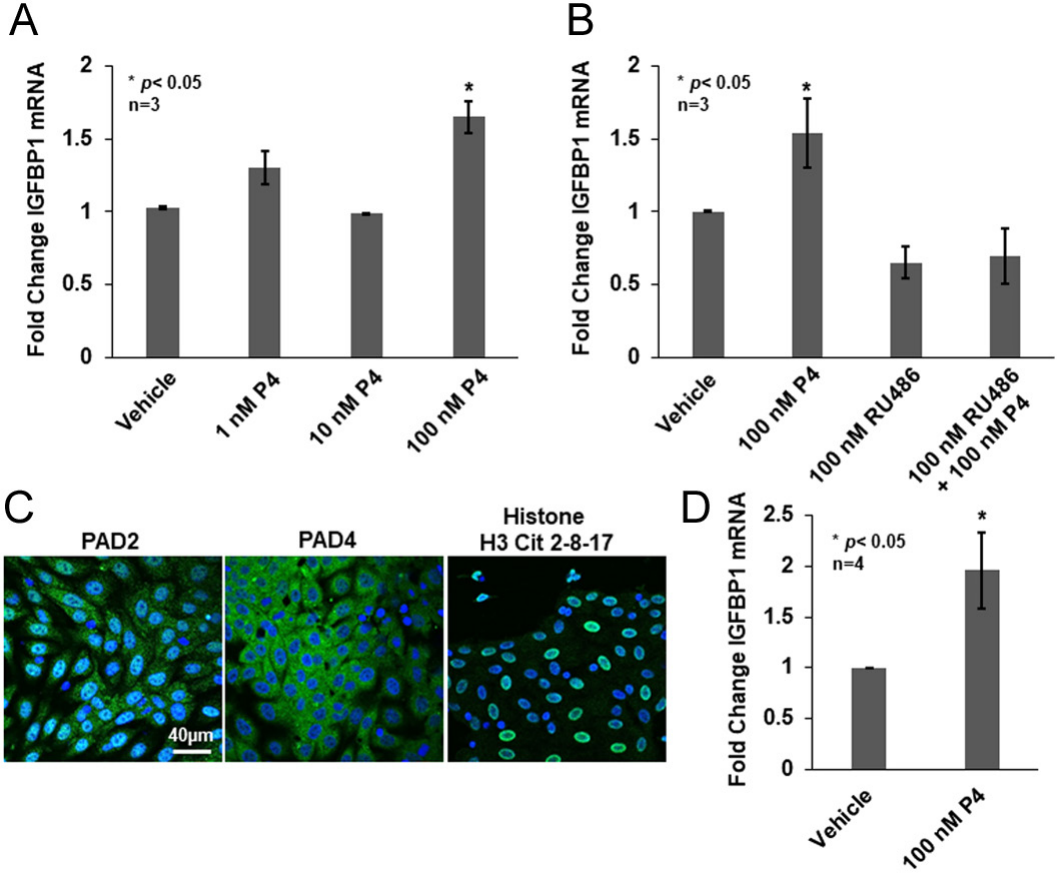
Progesterone stimulates IGFBP1 mRNA expression in OLE and ovine uterine primary epithelial cells. (A) OLE cells were treated with vehicle (DMSO) or with 1, 10, and 100 nM P4 for 2 h. Total RNA was purified, reverse transcribed, and the resulting cDNA examined by qPCR with intron spanning primers for IGFBP1 and GAPDH as the reference gene control. All values are expressed as means ± s.e.m. Means were separated using Student–Newman–Keuls (SNK) (*n* = 3, *P < 0.05). (B) OLE cells were treated with vehicle (Etoh) or 100 nM RU486 for 1 h followed by 100 nM P4 for an additional 2 h. Total RNA was purified, reverse transcribed, and the resulting cDNA examined by qPCR with intron spanning primers for IGFBP1 and GAPDH as the reference gene control. All values are expressed as means ± s.e.m. Means were separated using SNK (*n* = 3, *P < 0.05). (C) Ewe primary uterine epithelial cells were grown on glass bottom dishes overnight then fixed, permeabilized, and examined by immunocytochemistry using anti-PAD2, anti-PAD4, and anti-histone H3Cit 2, 8, 17 antibodies (green) and stained with DAPI (blue). Cells were imaged using a Zeiss LSM 710 confocal microscope using a 40× objective and scale bar is 40 µm. (D) Ewe primary uterine epithelial cells were grown overnight then the following morning treated with vehicle or 100 nM P4 for 2 h. Total RNA was purified from primary cells and reverse transcribed to cDNA. The cDNA was examined by qPCR with intron spanning primers for IGFBP1 and GAPDH as the reference gene control. All values are expressed as means ± s.e.m. Means were separated using a one-tailed paired *t*-test with * indicating significant differences (*n* = 4, *P < 0.05).

We previously found that PADs regulate basal IGFBP1 mRNA expression in OLE cells (Young *et al.* 2017). Since P4 stimulates *IGFBP1* expression in other models, we first tested if this also occurs in OLE cells. OLE cells were serum-starved overnight then treated with vehicle or 1, 10, and 100 nM P4 for 2 h. RNA was purified, reverse transcribed, and the resulting cDNA was analyzed by qPCR using primers specific for IGFBP1 with GAPDH serving as an endogenous control. Our results show a significant 1.6-fold increase in IGFBP1 mRNA following 2 h of 100 nM P4 treatment compared to vehicle-treated controls (Fig. 1A). Following the same paradigm, we next investigated whether the P4-induced increase in IGFBP1 mRNA expression is mediated by a PR. OLE cells were pre-treated with 100 nM RU486 for 1 h then stimulated with 100 nM P4 for an additional 2 h. While P4 alone stimulates an increase in expression, pre-treatment with a RU486 significantly reduces IGFBP1 mRNA levels indicating a PR-mediated mechanism (Fig. 1B).

In OLE cells, 100 nM P4 stimulates a significant increase in expression of *IGFBP1*, thus, we next validated this dose using ewe primary uterine luminal epithelial cells (Johnson *et al.* 1999). First, we confirmed that PAD2, PAD4, and citrullinated histones are present in dispersed ewe primary uterine luminal epithelial cells. Cells were grown overnight, fixed, and examined by immunocytochemistry (ICC) using anti-PAD2, anti-PAD4, and anti-histone H3Cit 2,8,17 antibodies. Cells were imaged using confocal laser scanning microscopy with a 40× objective. Both PAD2 and PAD4 staining is observed in the primary cells; however, PAD2 appears to have stronger nuclear localization compared to PAD4 (Fig. 1C). Imaging studies also detected strong histone H3Cit 2,8,17 staining in the nuclei of ewe primary uterine luminal epithelial cells. This PAD expression and citrullinated histone profile were also observed in our previous studies examining the OLE cell line and ewe caruncle histological tissue sections (Young *et al.* 2017). Lastly, we treated ewe primary uterine luminal epithelial cells with 100 nM P4 for 2 h and then quantified IGFBP1 mRNA expression. Our results show a significant two-fold increase in IGFBP1 mRNA following 100 nM P4 treatment recapitulating our OLE studies suggesting that this dose elicits a similar response in ewe primary uterine luminal epithelial cells (Fig. 1D).

### Progesterone stimulates PAD-catalyzed citrullination to regulate IGFBP1 mRNA expression

**Figure 2:**
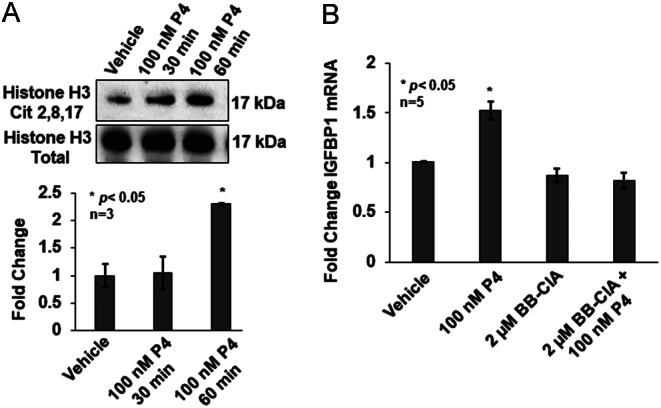
Progesterone stimulates PAD-catalyzed citrullination to regulate IGFBP1 mRNA expression. (A) OLE cells were stimulated with vehicle (DMSO) or 100 nM progesterone for 30 or 60 min. After cell lysis, histones were purified and equal amounts examined by Western blot. Membranes were probed with an anti-histone H3Cit 2, 8, 17 antibody or anti-total histone H3 as a loading control. The top panel shows a representative Western blot, while the bottom graph represents the quantification of multiple Western blots using BioRad Image Lab 4.0. Data are presented as means ± s.e.m. and separated using SNK (*n* = 3, *P < 0.05). (B) OLE cells were pretreated with vehicle (DMSO) or 2 μM BB-ClA for 3 h followed by stimulation with 100 nM P4 for 2 h. Total RNA was purified, reverse transcribed, and then cDNA was examined by qPCR with intron spanning primers for IGFBP1 and GAPDH as the reference gene control. All values are expressed as means ± s.e.m. Means were separated using SNK (*n* = 5, *P < 0.05).

We next investigated whether the P4-induced increase in IGFBP1 mRNA expression is mediated by PAD-catalyzed citrullination. To test this possibility, OLE cells were treated with vehicle or 100 nM P4 for 30 and 60 min. Following treatment, histones were isolated and equal concentrations were examined by Western blot. Membranes were probed with an anti-histone H3Cit 2,8,17 antibody and total histone H3 as the loading control. A representative Western blot illustrates that 60 min of 100 nM P4 treatment increases histone citrullination as compared to 60 min vehicle-treated controls (Fig. 2A). Quantification of multiple blots shows that P4 significantly increases histone H3 citrullination of arginine residues 2, 8, and 17 by greater than two-fold following 60 min of treatment as compared to vehicle alone (*n* = 3, *P < 0.05) (Fig. 2A). These results indicate that 100 nM P4 rapidly stimulates PAD-catalyzed histone citrullination in OLE cells.

These findings led us to hypothesize that P4 induces PAD-catalyzed citrullination to stimulate *IGFBP1* gene expression. To test this, we examined the expression of IGFBP1 mRNA in OLE cells following pre-treatment with DMSO or 2 μM BB-ClA for 3 h as previously described (Young *et al.* 2017) then treated for an additional 2 h with 100 nM P4 or vehicle. After treatment, RNA was purified, reverse transcribed, and resulting cDNA was analyzed by qPCR using primers specific for IGFBP1 with GAPDH serving as an endogenous control. Our results show a significant 1.5-fold increase in IGFBP1 mRNA following 2 h of P4 treatment; however, there is no P4-induced increase in IGFBP1 mRNA expression when cells are pre-treated with BB-ClA (*n* = 5, *P < 0.05) (Fig. 2B). This result suggests that P4 stimulates PAD-catalyzed citrullination to regulate IGFBP1 mRNA expression in OLE cells.

### Progesterone increases citrullinated histone H3 residues 2, 8, and 17 associated with the ovine IGFBP1 gene promoter

**Figure 3:**
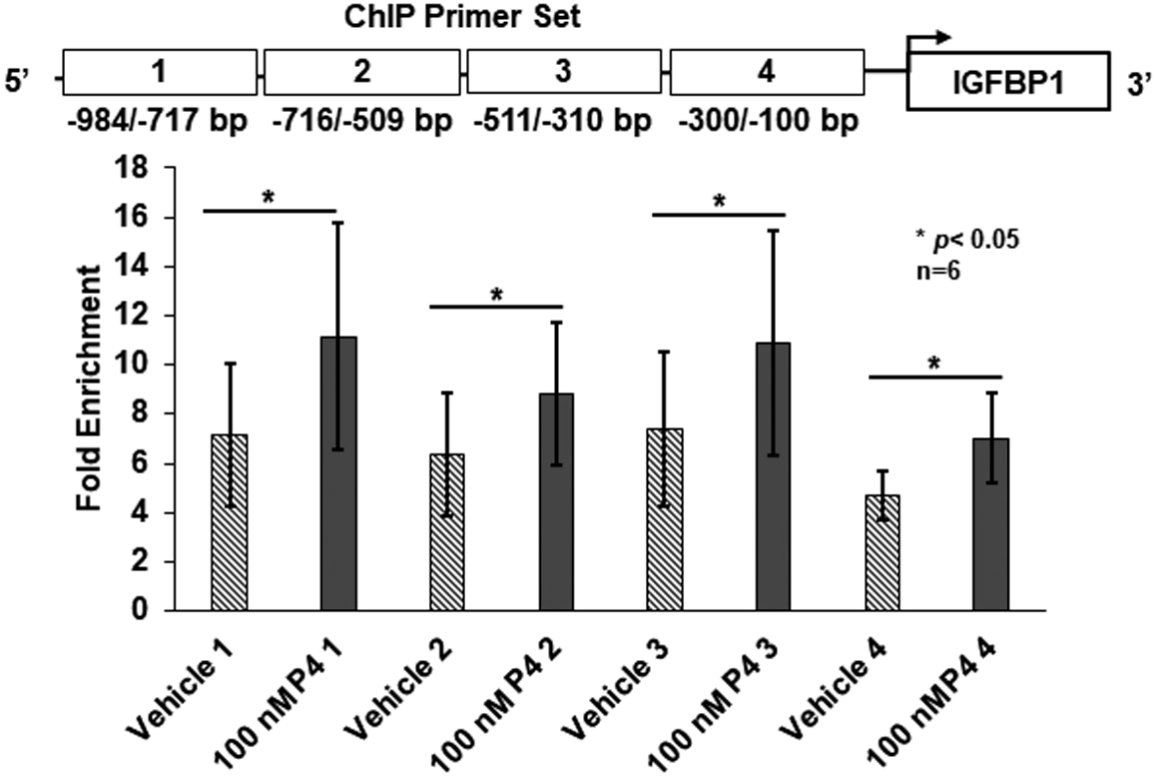
Progesterone increases citrullinated histone H3 residues 2, 8, and 17 associated with the ovine IGFBP1 gene promoter. OLE cells were treated with vehicle or 100 nM P4 for 60 min. Cross-linked histone-DNA complexes were immunoprecipitated with an anti-histone H3Cit 2,8,17 antibody, anti-histone H3 antibody (positive control), or nonspecific IgG (negative control). After reversing cross-links, DNA was purified and examined by qPCR with primers that scanned the proximal ovine IGFBP1 gene promoter. Results were analyzed using the fold enrichment method, all values are means ± s.e.m., and means were separated using a one-tailed paired *t*-test (*n* = 6, *P < 0.05).

Since PAD inhibition blocks P4-induced IGFBP1 mRNA expression, we investigated if citrullinated histones are directly associated with the ovine *IGFBP1* gene promoter. Chromatin immunoprecipitation (ChIP) was performed using OLE cells that were treated with either vehicle or 100 nM P4 for 60 min. Post-treatment, OLE chromatin was immunoprecipitated with the anti-histone H3Cit 2,8,17 antibody and analyzed by qPCR using four sets of primers that scan approximately 200 bps. The four primer sets cover approximately 1000 bp upstream of the ovine *IGFBP1* gene transcriptional start site. Our ChIP results show that P4 treatment significantly increases enrichment of citrullinated histone H3 residues directly associated with the *IGFBP1* gene promoter in all primer sets as compared to vehicle-treated cells (*n* = 6, *P < 0.05) (Fig. 3). These results are the first to find that P4 significantly increases histone citrullination at the ovine *IGFBP1* gene promoter.

### Progesterone rapidly stimulates an increase in intracellular calcium via L-type calcium channels in OLE cells

**Figure 4:**
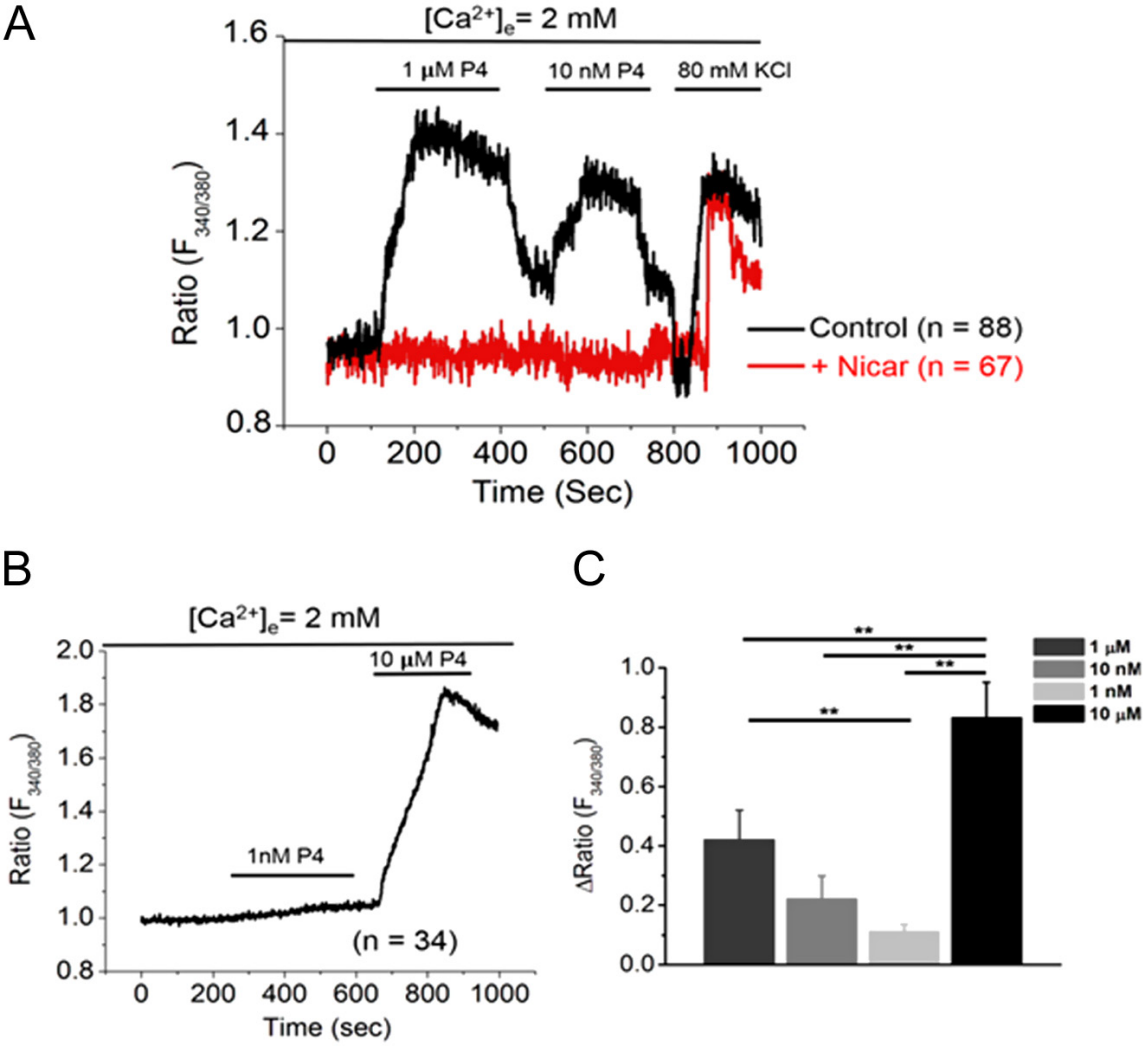
Progesterone stimulates a rapid increase in intracellular calcium via L-type calcium channels in OLE cells. OLE cells were plated on 25 mm circular coverslips and incubated overnight. The following morning, cells were incubated with 2 μM Fura-2AM for 1 h at room temperature. The cells were then mounted in a flow cell with normal extracellular saline (NES) buffer. (A) Cells were treated with flow through of 1 µM P4 for 100–400 s, 10 nM P4 for 500–700 s, and with 80 mM KCl at 800 s with NES washes in between each treatment (*n* = 88 cells quantified). In parallel, another coverslip of cells was pretreated with 10 nM nicardipine for 30 min and then subjected to the same P4 treatment protocol (*n* = 67 cells quantified). (B) Cells were treated with 1 nM P4 for 100–400 s and with 10 µM P4 for 600–800 s followed by a wash at 1000 s (*n* = 34 cells quantified). (C) The bar graph represents the analysis of the mean change in 340/380 ratio for all P4 treatments: 1 µM (*n* = 88), 10 nM (*n* = 88), 1 nM (*n* = 34), 10 µM (*n* = 34). Statistical significance was calculated using one-way ANOVA followed by Student’s *t*-test. Data are expressed as mean + s.e.m. and ** represents statistical significance (*P* < 0.01). Analyzed data were plotted using Microcal Origin 2020 software.

Our previous work in the gonadotropin-derived LβT2 cell line discovered that activation of L-type calcium channels is necessary for PAD2 nuclear translocation (Khan *et al.* 2016). This led us to hypothesize that P4 induces a rapid calcium influx via L-type calcium channels in OLE cells. OLE cells were loaded with 2 μM fluorescent calcium indicator Fura-2AM for 1 h at room temperature, then mounted in a flow cell with normal extracellular saline (NES) buffer. Cells were treated by flow-through of 1 µM P4 from 100 to 400 s, 10 nM P4 from 500 to 700 s, and 80 mM KCl at 800 s (*n* = 88 cells quantified) (Fig. 4A, black trace). Between each treatment, cells were washed for 100 s with NES to flush the previous treatment. KCl was given as a positive control as it induces massive calcium influx in live cells. In parallel, a second coverslip of cells was pretreated with 10 nM of the L-type calcium channel blocker nicardipine for 30 min followed by the same treatments described previously (*n* = 67 cells quantified) (Fig. 4A, red trace) (Kasai *et al.* 1995). An additional coverslip of cells was treated with 1 nM P4 for 100–400 s and then with 1 µM P4 for 400–700 s (*n* = 34 cells quantified) (Fig. 4B). Our results show a P4 dose-dependent increase in the 340/380 fluorescence ratio in OLE cells indicating a rapid calcium influx following treatment (Fig. 4C). Moreover, when the OLE cells are pretreated with nicardipine, the 340/380 fluorescence ratio does not increase following P4 treatment, indicating that extracellular calcium influx occurs via L-type calcium channels.

### PAD2 nuclear translocation and histone citrullination are dependent on progesterone-induced activation of L-type calcium channels in OLE cells

**Figure 5:**
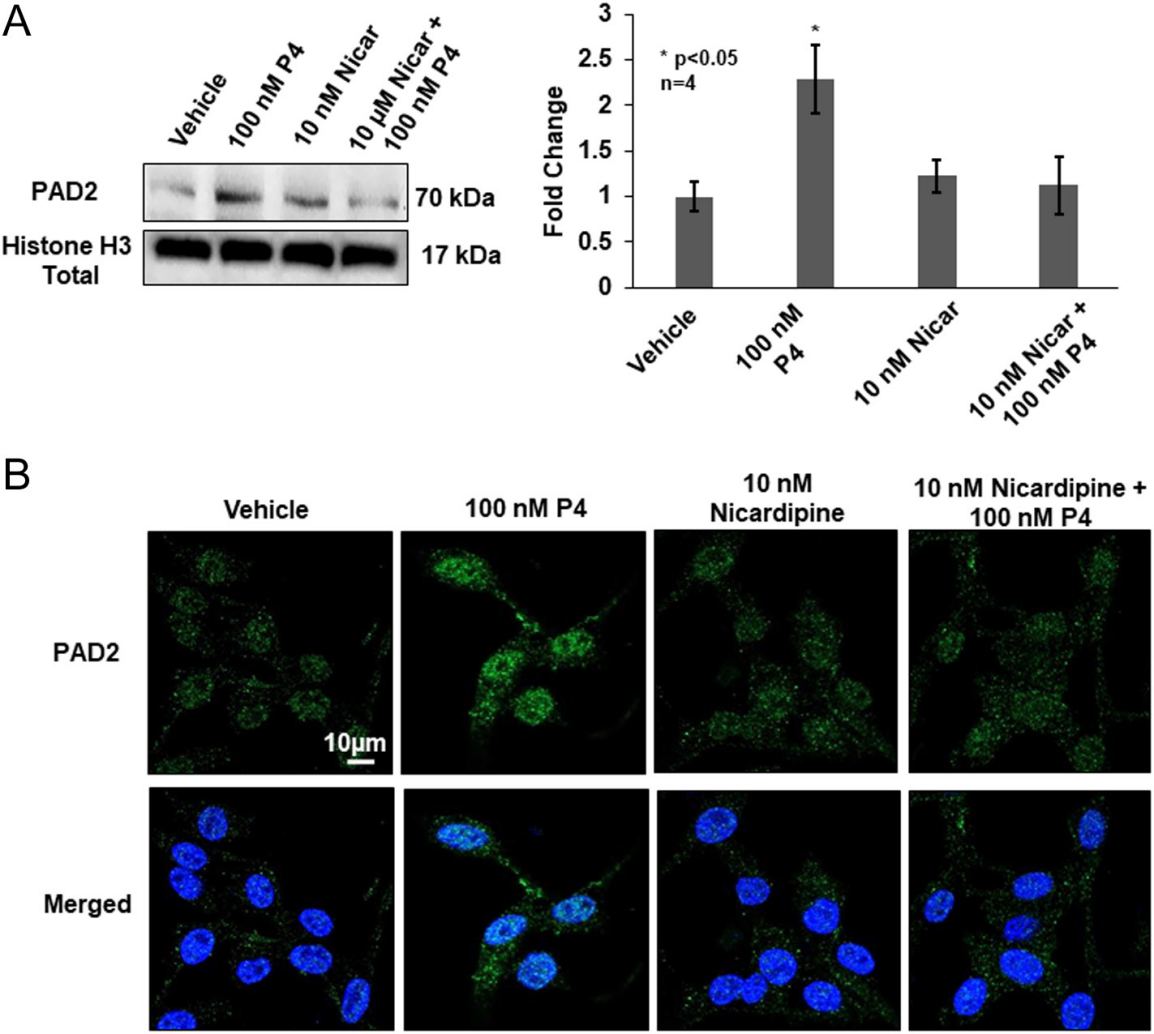
Progesterone stimulates calcium influx via L-type calcium channels to mediate PAD2 nuclear translocation. (A) OLE cells were pre-treated with vehicle (DMSO) or 10 nM nicardipine for 30 min then stimulated with vehicle (DMSO) or 100 nM P4 for an additional 30 min. Following cell lysis, chromatin-associated nuclear proteins were isolated and equal amounts were examined by Western blot. Membranes were probed with an anti-PAD2 antibody or anti-total histone H3 as a loading control. The left panel shows a representative Western blot, while the graph on the right illustrates the quantification of multiple Western blots using BioRad Image Lab 4.0. Data are presented as means ± s.e.m. and separated using SNK (*n* = 4, *P < 0.05). (B) OLE cells were grown on glass bottom dishes overnight then fixed, permeabilized, and examined by immunocytochemistry using an anti-PAD2 antibody (green) and stained with DAPI (blue). Cells were imaged using a Zeiss LSM 980 confocal microscope using a 63× objective and scale bar is 10 µm.

PAD2 nuclear translocation and catalytic activity are both calcium-dependent (Arita *et al.* 2004, Mondal & Thompson 2019, Zheng *et al.* 2019). Thus, we hypothesized that P4-induced calcium influx through L-type calcium channels is necessary for PAD2 nuclear translocation and subsequent histone citrullination. To test this hypothesis, OLE cells were pretreated with DMSO or 10 nM nicardipine for 30 min followed by stimulation with 100 nM P4 or vehicle for an additional 30 min. Following treatment, chromatin-associated proteins were isolated and equal concentrations were examined by Western blot using an anti-PAD2 antibody. A representative Western blot shows that 30 min of P4 treatment increases PAD2 nuclear translocation as compared to vehicle-treated controls; however, pretreatment with nicardipine attenuates PAD2 nuclear localization (Fig. 5A). Quantification of multiple blots (*n* = 4, *P < 0.05) supports that P4-induced PAD2 nuclear translocation is significantly blunted by blocking calcium influx through L-type calcium channels in OLE cells. These results were corroborated by performing immunocytochemistry on OLE cells treated as described previously. 100 nM P4 treatment of OLE cells for 30 min results in an increase in PAD2 staining in the nucleus, but this is blunted by the pretreatment with 10 nM nicardipine (Fig. 5B).

**Figure 6:**
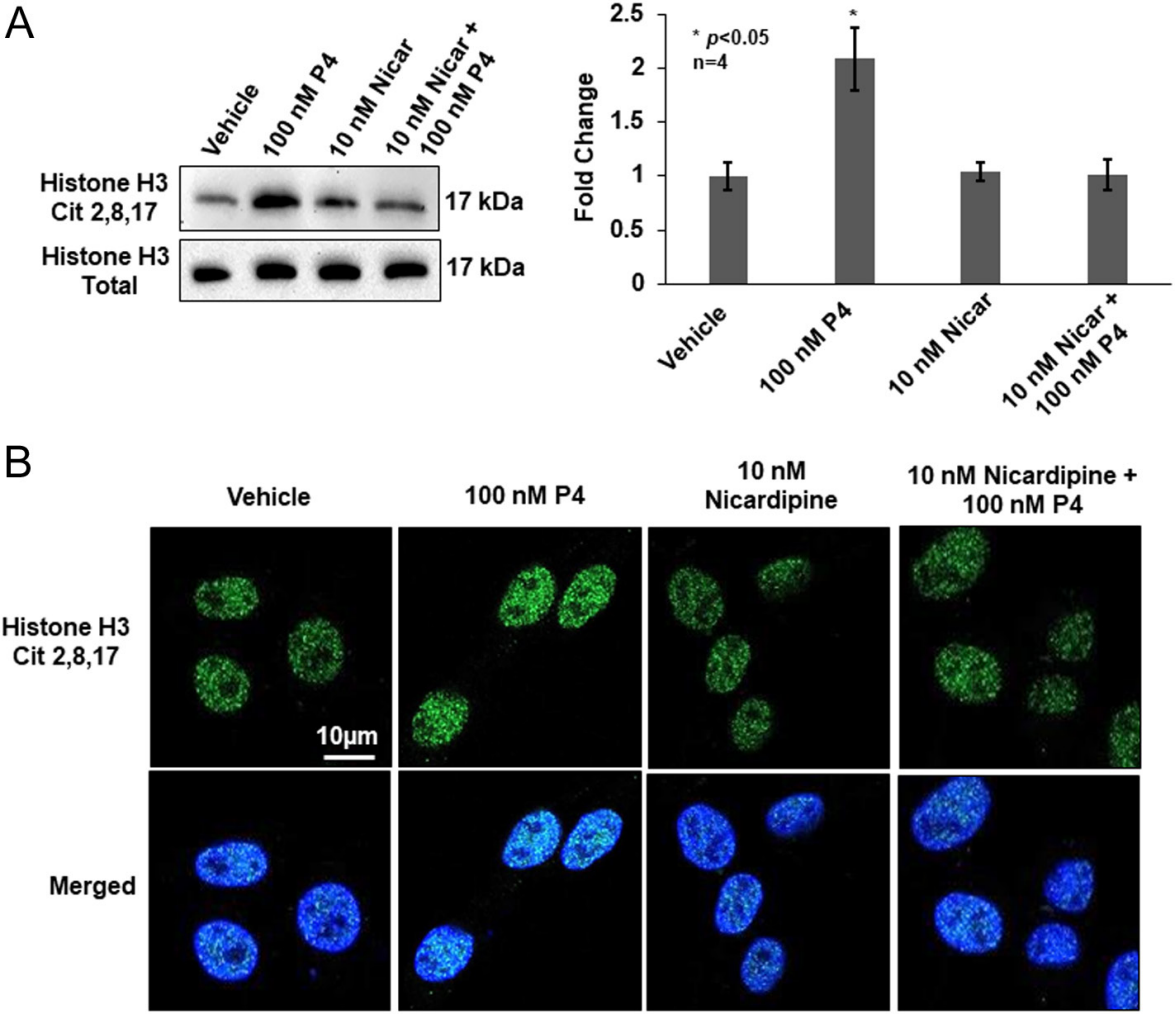
Progesterone stimulates calcium influx via L-type calcium channels resulting in increased histone H3 citrullination. (A) OLE cells were pretreated with vehicle (DMSO) or 10 nM nicardipine for 30 min then stimulated with vehicle (DMSO) or 100 nM P4 for an additional 60 min. Following cell lysis, chromatin-associated nuclear proteins were isolated and equal amounts were examined by Western blot. Membranes were probed with an anti-Histone H3Cit 2,8,17 antibody or anti-total histone H3 as a loading control. The top left panel shows a representative Western blot, while the graph to the right illustrates the quantification of multiple Western blots using BioRad Image Lab 4.0. Data are presented as means ± s.e.m. and separated using SNK (*n* = 4, *P < 0.05). (B) OLE cells were grown on glass bottom dishes overnight then fixed, permeabilized, and examined by immunocytochemistry using an anti-Histone H3Cit 2,8,17 antibody (green) and stained with DAPI (blue). Cells were imaged using a Zeiss LSM 980 confocal microscope using a 63X objective and scale bar is 10 µm.

Once PAD2 translocates to the nucleus, a corresponding increase in histone citrullination should occur. We tested this idea by pretreating OLE cells for 30 min with 10 nM nicardipine then stimulating them with 100 nM P4 for 60 min when histone H3Cit 2,8,17 levels are maximal (Fig. 2A). After purification, equal concentrations of histones were examined by Western blot, and membranes were probed with the anti-histone H3Cit 2,8,17 antibody and total histone H3 antibody as a loading control. A representative Western blot indicates that 60 min of P4 treatment increases histone citrullination as compared to vehicle-treated controls; however, pre-treatment with nicardipine attenuates this increase (Fig. 6B). Quantification of multiple blots (*n* = 4, *P < 0.05) indicates that P4-induced histone citrullination is significantly blunted by blocking calcium influx through L-type calcium channels. This finding was corroborated by performing immunocytochemistry on OLE cells treated as described previously. Treatment of OLE cells for 60 min with 100 nM P4 results in an increase in H3Cit 2,8,17 staining in the nucleus, but this is blunted by the pretreatment with 10 nM nicardipine (Fig. 6B). Taken together, our results suggest that P4 stimulates a rapid calcium influx through L-type calcium channels that are important for PAD2 nuclear translocation and histone citrullination.

## Discussion

In women, pregnancy loss or miscarriage occurs in approximately 20% of all pregnancies with the majority occurring before the 12th week of gestation (Dante *et al.* 2013). Embryo loss in early pregnancy is also an important consideration in agricultural animals (Diskin *et al.* 2006, Dixon *et al.* 2007, Kwak-Kim *et al.* 2010). The uterine milk or histotroph, which is comprised of numerous molecules secreted by uterine glandular epithelial cells, is essential to nourish the early embryo, facilitate placental development, and for blastocyst survival and growth (Spencer *et al.* 2004). In particular, IGFBP1 facilitates migration and attachment of the trophectoderm in multiple species (Gleeson *et al.* 2001, Brooks *et al.* 2014). Our previous work shows that basal *IGFBP1* expression in OLE cells is regulated by histone citrullination (Young *et al.* 2017). Since P4 is known to stimulate *IGFBP1* expression, herein we investigated the mechanism by which P4 activates PAD-catalyzed histone citrullination to regulate IGFBP1 expression.

For these studies, we used OLE cells that were originally isolated from uterine epithelium collected on the 5th day of the ewe estrous cycle (Johnson *et al.* 1999). Our previous studies found that OLE cells express PAD enzymes and contain citrullinated histones (Young *et al.* 2017). We chose to examine mRNA expression after 2 h of P4 treatment since *IGFBP1* can be rapidly and dynamically regulated (Tazuke *et al.* 1998, Fowler *et al.* 2000). IGFBP1 mRNA expression in OLE cells was first examined following treatment with increasing concentrations of P4. 100 nM P4 stimulates a significant increase in IGFBP1 mRNA expression in OLE cells and in ewe primary uterine epithelial cells. Although this is higher than found in ewe serum, this concentration has been used to treat OLE cells and in other model systems (Banu *et al.* 2010, Goddard *et al.* 2014, Kavlashvili *et al.* 2016).

Work from our lab found that gonadotropin-releasing hormone (GnRH) induces calcium influx through L-type calcium channels resulting in PAD2 nuclear translocation in gonadotropin-derived LβT2 cells (Edwards *et al.* 2016, Khan *et al.* 2016). PAD2 does not contain a classical nuclear localization sequence, but Zheng *et al.* found that PAD2 nuclear translocation is a calcium-dependent process (Zheng *et al.* 2019). The binding of calcium to PAD2 weakens its interaction with annexin 5, a phospholipid-binding protein, allowing increased association with RanGDP which facilitates nuclear translocation (Zheng *et al.* 2019). Based on these findings, we tested whether P4 stimulates a rapid calcium influx via activation of L-type calcium channels in OLE cells thereby stimulating PAD2 nuclear translocation. Our calcium imaging data clearly shows that P4 stimulates rapid calcium influx via L-type calcium channels. However, we cannot rule out the contribution of intracellular calcium mobilization on PAD2 translocation, a possibility that will require further investigation. Collectively, our results suggest that P4 initiates a rapid calcium influx in OLE cells, which causes PAD2 nuclear translocation which ultimately results in increased histone citrullination.

Once in the OLE nucleus, PADs citrullinated histone H3 arginine residues 2, 8, and 17 with maximal levels occurring after 60 min of P4 treatment. PADs hydrolyze the positive guanidinium group of arginine residues on histones converting arginine to neutral citrulline, which modifies chromatin structure to alter gene expression (Hagiwara *et al.* 2002, Wang *et al.* 2009, Tanikawa *et al.* 2012). Pretreating OLE cells with 2 µM BB-ClA, which binds covalently to the PAD enzyme active site, blunts the P4-induced increase in IGFBP1 mRNA (Horibata *et al.* 2015, Knight *et al.* 2015). Although P4 stimulates the expression of critical endometrial genes that code for proteins that comprise the histotroph such as *IGFBP1*, the underlying epigenetic mechanisms remain poorly characterized (Spencer *et al.* 2016). Our ChIP results indicate that the proximal promoter region of the ovine *IGFBP1* gene is enriched with citrullinated histones following P4 treatment compared to vehicle-treated controls. In addition to histone citrullination regulating expression, Tamura *et al.* found an increase in acetylation of histone H3 lysine 27 associated with the *IGFBP1* gene promoter during decidualization (Tamura *et al.* 2018). In women, the *IGFBP1* gene promoter contains a progesterone response element (PRE) (Gao *et al.* 1999); yet, to the best of our knowledge, the ovine *IGFBP1* gene does not contain a PRE. Sequence analysis of the ovine *IGFBP1* gene promoter region shows putative binding sites for the forkhead winged-helix (FOX) family of transcription factors. In human endometrial cells, FOXO1A interacts with the nPR to induce expression of the *IGFBP1* gene (Kim *et al.* 2005). Our results suggest that nPRs may be interacting with a number of transcription factors such as FOXO1A that bind at multiple sites across the proximal ovine *IGFBP1* gene promoter. Clearly, additional studies are necessary to determine how the ovine *IGFBP1* gene is regulated by P4 in the absence of a PRE.

In uterine tissue, L-type calcium channels are present in the myometrium and mediate the calcium influx required for parturition (Collins *et al.* 2000). In human endometrial cells, channel blockers reduce intracellular calcium levels resulting in changes in the expression of decidualization and glandular maturation genes. Specifically, blocking calcium influx in human endometrial cells results in a decrease in IGFBP1 levels supporting the idea that a calcium-dependent mechanism regulates *IGFBP1* expression (Kusama *et al.* 2015). Yet, our results pose an interesting mechanistic question regarding how P4 activates L-type calcium channels in OLE cells irrespective of P4’s role in stimulating *IGFBP1* gene expression. In the gonadotropin-derived αT3-1 cell line, GnRH treatment, acting through the GnRH receptor (GnRHR), requires protein kinase C and the cytoskeletal filament actin to activate L-type calcium channels. This activation generates localized subplasma membrane sites of calcium influx, termed 'calcium sparklets' (Dang *et al.* 2014). A similar high calcium microdomain localized close to intracellular ion channels has been hypothesized to mediate the calcium environment necessary for full PAD catalytic activation. It is currently unclear if the rapid calcium influx in OLE cells following P4 stimulation is mediated by the nuclear or membrane receptor or perhaps a combination of both. Although P4 is well-documented to increase intracellular calcium rapidly in several cell types, the roles of nuclear and membrane PRs are still unresolved (Wasserman *et al.* 1980, Thomas & Meizel 1989). For example, the nPR can interact with intracellular signaling machinery to mediate non-genomic effects of P4, while other studies have shown mPRs can alter calcium signaling (Ashley *et al.* 2006, Boonyaratanakornkit & Edwards 2007, Lee *et al.* 2010, Singh *et al.* 2013). Further studies are clearly necessary to determine the mechanism by which P4 activates L-type channels to regulate calcium influx in OLE cells.

In summary, our work shows that P4 initiates a rapid calcium influx through L-type calcium channels that stimulate PAD2 nuclear translocation and histone citrullination to help regulate *IGFBP1* expression. These studies demonstrate that PADs play an important role in epigenetic gene regulation in uterine luminal epithelium and further, our knowledge of transcriptional regulation of the important histotroph gene *IGFBP1*. Understanding the epigenetic mechanisms regulating histotroph genes like *IGFBP1* may lead to future fertility therapeutics to prevent recurrent pregnancy loss in multiple species.

## Declaration of interest

The authors declare no conflict of interest that could be perceived as prejudicing the impartiality of the research reported.

## Funding

Research reported in this publication was supported by the National Institute of General Medical Sciences of the National Institutes of Health under the Award Number P20GM103432 (B D C), Eunice Kennedy Shriver National Institute of Childhood Health and Disease R21HD090541 (B D C and A M N), NIH NIGMS P20-121310-03 (B T), and in part by R35 GM118112 (P R T). The content is solely the responsibility of the authors and does not necessarily represent the official views of the National Institutes of Health.

## Author contribution statement

Conceptualization was done by C H Y, A M N, B T and B D C; methodology was given by C H Y, B T and B D C; validation was done by C H Y and A M; formal analysis was done by C H Y, B S, A M and S B D; investigation was carried out by C H Y and B D C; resources were provided by V V N and P R T; data curatio was done by B D C; writing – original draft preparation was done by C H Y; writing – review and editing was done by C H Y and B D C; visualization was done by C Y H, B S, S B D and B D C; supervision and project administration were done by B D C; funding acquisition was done by B D C and P R T. All authors have read and agreed to the published version of the manuscript.
